# *Fusarium* *culmorum* Produces NX-2 Toxin Simultaneously with Deoxynivalenol and 3-Acetyl-Deoxynivalenol or Nivalenol

**DOI:** 10.3390/toxins14070456

**Published:** 2022-07-02

**Authors:** Simon Schiwek, Mohammad Alhussein, Charlotte Rodemann, Tuvshinjargal Budragchaa, Lukas Beule, Andreas von Tiedemann, Petr Karlovsky

**Affiliations:** 1 Institute for Plant Protection in Field Crops and Grassland, Julius Kuehn-Institute, D-38104 Braunschweig, Germany; 2 Molecular Phytopathology and Mycotoxin Research, University of Goettingen, D-37077 Goettingen, Germany; mohammad.alhussein@uni-goettingen.de; 3 Plant Phytopathology and Crop Protection, University of Goettingen, D-37077 Goettingen, Germany; charlotte.rodemann@uni-goettingen.de (C.R.); atiedem@gwdg.de (A.v.T.); 4 Department of Bioorganic Chemistry, Leibniz Institute for Plant Biochemistry, D-06120 Halle, Germany; tuvshinjargal.budragchaa@ipb-halle.de; 5 Plant Analysis and Stored Product Protection, Institute for Ecological Chemistry, Julius Kuehn-Institute, D-14195 Berlin, Germany; lukas.beule@julius-kuehn.de

**Keywords:** NX-2, NX toxins, *Fusarium culmorum*, 3-actyl-deoxynivalenol, nivalenol, trichothecenes, chemotype

## Abstract

*Fusarium* *culmorum* is a major pathogen of grain crops. Infected plants accumulate deoxynivalenol (DON), 3-acetyl-deoxynivalenol (3-ADON), or nivalenol (NIV), which are mycotoxins of the trichothecene B group. These toxins are also produced by *F.* *graminearum* species complex. New trichothecenes structurally similar to trichothecenes B but lacking the carbonyl group on C-8, designated NX toxins, were recently discovered in atypical isolates of *F.* *graminearum* from North America. Only these isolates and a few strains of a yet to be characterized *Fusarium* species from South Africa are known to produce NX-2 and other NX toxins. Here, we report that among 20 *F. culmorum* strains isolated from maize, wheat, and oat in Europe and Asia over a period of 70 years, 18 strains produced NX-2 simultaneously with 3-ADON and DON or NIV. Rice cultures of strains producing 3-ADON accumulated NX-2 in amounts corresponding to 2–8% of 3-ADON (1.2–36 mg/kg). A strain producing NIV accumulated NX-2 and NIV at comparable amounts (13.6 and 10.3 mg/kg, respectively). In *F. graminearum*, producers of NX-2 possess a special variant of cytochrome P450 monooxygenase encoded by *TRI1* that is unable to oxidize C-8. In *F.* *culmorum*, producers and nonproducers of NX-2 possess identical *TRI1*; the reason for the production of NX-2 is unknown. Our results indicate that the production of NX-2 simultaneously with trichothecenes B is a common feature of *F.* *culmorum*.

## 1. Introduction

*Fusarium* head blight (FHB) is a cosmopolitan disease of small-grain cereals caused by several *Fusarium* species with a high economic impact [1]. *Fusarium graminearum* and *F. culmorum* belong to the predominant agents of FHB [2]. Infection of grain crops with *Fusarium* spp. causes yield losses and contamination of grains with toxic metabolites (mycotoxins), impairing food safety [3,4].

*Fusarium culmorum* belongs to the *F. sambucinum* species complex [5]. It infects a wide range of grain crops such as wheat [2,6], maize [7,8,9], barley [10,11], oat [10], triticale [11], and rye [11]. *Fusarium culmorum* typically co-occurs with other *Fusarium* species. In most reports, the incidence of *F. culmorum* in crops was second to *F. graminearum*, but in some crops, growing regions, and years, *F. culmorum* dominated [2,6,10]. Infected plant material often contains trichothecene mycotoxins deoxynivalenol (DON) and its derivatives 3-acetyl-deoxynivalenol (3-ADON) and 15-acetyl-deoxynivalenol (15-ADON), nivalenol (NIV) and its derivative fusarenon X (4-acetyl-nivalenol), and resorcylic acid lactone zearalenone (ZEN) [4,12].

Biosynthesis of trichothecenes in *Fusarium* spp. is encoded by *TRI* genes, comprising a core *TRI* cluster and one or two additional loci, depending on the species [13]. According to the dominant trichothecene produced, *Fusarium* strains producing trichothecenes B are partitioned into the 3-ADON chemotype, the 15-ADON chemotype, and the NIV chemotype [13,14]. 

The distribution of chemotypes among wheat-producing regions exhibits a strong geographic pattern, with most areas dominated by either the 3-ADON or 15-ADON chemotype (reviewed in [15]; a succinct overview can also be found in the introduction of [16]). The 3-ADON chemotype in the USA was assumed to be introduced from Europe [17]. Some studies have not found any relationship between the chemotype and aggressiveness of *F. graminearum* [18,19], but other studies found the 3-ADON chemotype was more aggressive than the 15-ADON chemotype [20,21,22,23]. As several authors suggested [20,21], the discrepancy between earlier and later studies may be accounted for by the use of different inoculation methods because DON is not required for initial infection, but it facilitates the spread of the pathogen along the spike. The greater aggressiveness of the 3-ADON chemotype as compared to the 15-ADON chemotype is in line with a shift in *F. graminearum* populations from 15-DON to 3-ADON producers in North America over the last two decades [16,21]. In several studies on the population structure of *F. graminearum,* a fitness advantage of 3-ADON producers due to their higher aggressiveness was postulated and correlations with growth rates and spore production were determined. The reason for the greater aggressiveness of 3-ADON producers as compared to 15-ADON producers has rarely been addressed. A plausible hypothesis was coined by Poppenberger et al. [24] based on their finding that 3-ADON was protected against glucosylation by UDP-glucosyltransferase from *Arabidopsis thaliana* (see also Section 3.5).

The trichothecene chemotype has been monitored extensively in *F. graminearum* (e.g., [9,16,18,19,20,21,22,23,25]; reviewed in [15]). Fewer studies monitored the chemotype in *F. culmorum,* which only comprises the 3-ADON and NIV types [9,26,27,28]. Few studies in *F. culmorum* have monitored fusarenon X in pure cultures; cultures of NIV producers typically accumulated fusarenon X, some at higher and some at lower concentrations than NIV. In the past, chemotypes were often assigned according to polymorphisms in *TRI* genes [25], but frequently reported discrepancies between the chemotype prediction by PCR-RFLP and the results of chemical analysis have questioned this approach [14,16,29]. 

During an investigation of *F. graminearum* strains colonizing wheat heads in North America, a new type A trichothecene mycotoxin, the NX-2, was discovered [30]. The structure of NX-2 is similar to 3-ADON, but it lacks a carbonyl group at C-8. A deacetylated derivative of NX-2, called NX-3, has also been reported. Very recently, these metabolites have also been found in a yet to be characterized species of the *Fusarium sambucinum* species complex from South Africa [31]. The first strains of *F. graminearum* from North America producing NX toxins did not produce any trichothecene type B under laboratory conditions. The lack of known trichothecenes actually motivated the investigation that led to the discovery of NX toxins [30]. Strains producing NX-2 were originally found at a very low frequency, but a recent study by Lofgren et al. [32] estimated that 20% of the *F. graminearum* population in the USA produces NX-2. An even more recent study of *F. graminearum* strains in Canada [16] identified producers of NX-2 (which they designated 3ANX for consistency with the nomenclature of ADONs) among strains of *F. graminearum* assigned to the 15-ADON chemotype. Contrary to the reports that NX-2-producing strains from the USA have not produced DON, NIV, or their acetylated derivatives [30,33], most strains in their work [16] produced NX-2 simultaneously with 15-ADON.

Here, we report that most isolates of *F. culmorum* collected in Europe and Asia produce NX-2 toxin simultaneously with DON and 3-ADON or NIV. 

## 2. Results

### 2.1. Mycotoxin Production in Rice Cultures

**Figure 1:**
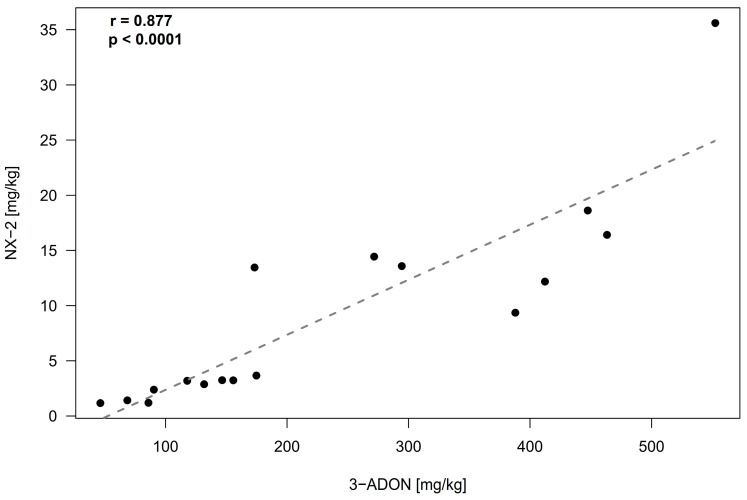
Relationship (Pearson correlation) between NX-2 and 3-ADON concentrations in rice cultures of *F. culmorum*. Each culture (Table 1) is represented by a single data point (black dots). Isolate 59.6st, which was assigned to the NIV chemotype, and isolates 227.2 and IPP0999, in which the concentrations of NX-2 were below the limit of detection, were excluded.

Our set of 20 *F. culmorum* strains consisted of 14 strains isolated from maize, wheat, and oat in Germany and 6 strains obtained from other laboratories and culture collections, which were isolated in different countries in a time span of 70 years. The analysis of rice culture extracts revealed an accumulation of NX-2 at concentrations larger than 1 mg/kg in 18 cultures (Table 1). All strains except one were primarily 3-ADON producers; one isolate was assigned to the NIV chemotype. The culture of this strain also accumulated fusarenon X, which is common in NIV producers. The concentration of 3-ADON in rice cultures of DON/ADON producers was high, exceeding 100 mg/kg in the cultures of 12 out of 20 strains. The concentrations of NX-2 and 3-ADON in the cultures of the strains of the 3-ADON chemotype were tightly correlated (Figure 1). NX-2 in these cultures accumulated to levels corresponding to 2–8% of 3-ADON. In the culture of the only isolate of the NIV chemotype (isolate 59.6st), the concentrations of NIV and NX-2 were comparable (Table 1).

### 2.2. Confirmation of the Structure of NX-2

**Figure 2:**
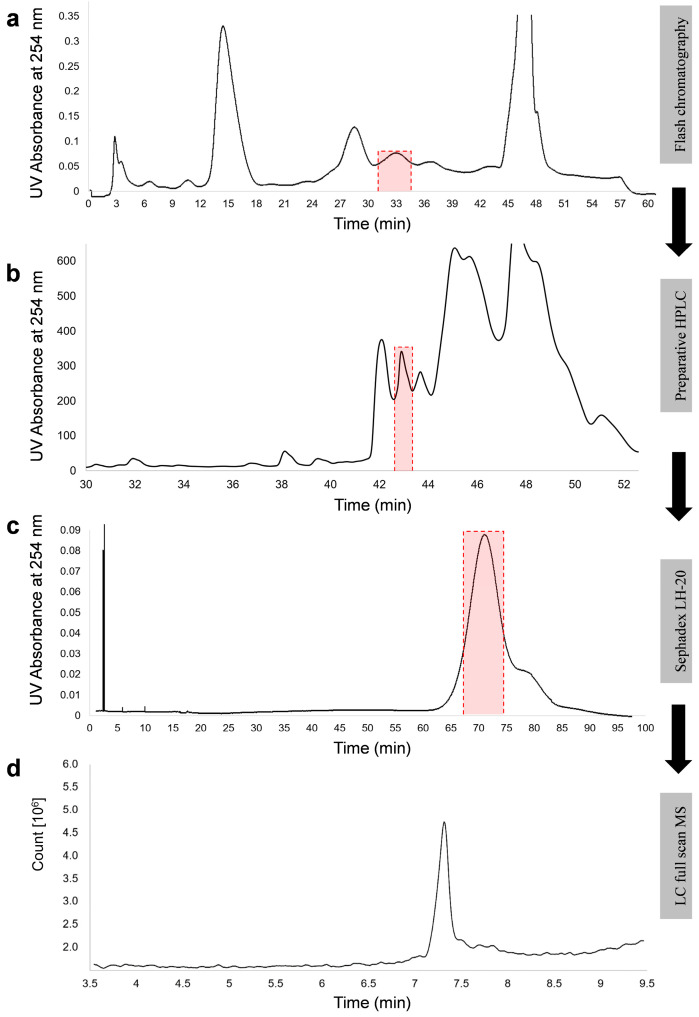
Purification of NX-2 toxin from rice culture of *F. culmorum* 240.2sp. Three-week-old rice cultures were extracted with methanol/water/acetic acid and putative NX-2 was enriched by chromatography on a C18 cartridge (**a**), polar-modified C18 column (**b**), and Sephadex LH-20 (**c**). The purity of the metabolite was established by HPLC-MS in a full-scan mode (**d**). Red boxes mark fractions collected for purification.

Putative NX-2 accumulating in rice cultures of *F. culmorum* was originally identified by comparing its retention time in HPLC and MS/MS fragmentation with data obtained with purified NX-2 from Prof. Franz Berthiller (BOKU, Vienna, Austria). Because the production of NX-2 by *F. culmorum* has not been reported before and an isomer of NX-2 could possess the same retention time and generate product ions with the same *m*/*z* values, we purified putative NX-2 from rice cultures of *F. culmorum* 240.2sp (Figure 2) to verify its structure. Approximately 5 mg of pure metabolite was obtained from 480 g of dry rice culture (see Section 5.4 for details). 

**Figure 3:**
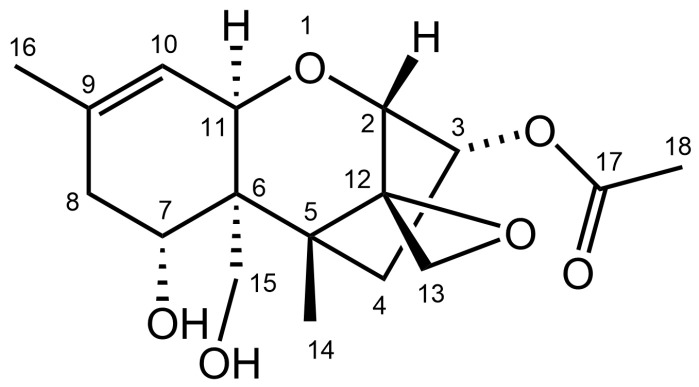
Structure of NX-2 toxin.

One- (^1^H and ^13^C) and two-dimensional (^1^H,^13^C-HSQC, ^1^H,^13^C-HMBC, ^1^H,^1^H-COSY) NMR spectroscopic analysis was performed on the purified metabolite (Supplementary Figures S1–S5). The spectroscopic data were in accordance with published data for NX-2 (Supplementary Table S1). The structure of NX-2 is shown in Figure 3.

### 2.3. Species Assignment and Investigation of Polymorphisms in TRI1

The examination of conidia assigned all isolates analyzed for trichothecene production (Table 1) to *Fusarium culmorum*. Morphological characterization was complemented by the analysis of the melting curves of amplicons of taxonomically informative genes encoding translation elongation factor 1α (TEF-*1α*) and the second largest component of the RNA polymerases II (*RPB2*) [34]. Species identification was further strengthened by the analysis of the full-length sequence (1753 nt) of the *TRI1* gene, which encodes cytochrome P450 monooxygenase catalyzing oxygenation of calonectrin on C-7 and C-8. The sequences of *TRI1* obtained from isolates of *F. culmorum* used in this study (GeneBank accession numbers OM144918 to OM144937) were aligned with a set of reference sequences (Supplementary Table S3) and used for phylogenetic analysis using the maximum-likelihood method. Separation of *F. culmorum* from other *Fusarium* species was highly supported, indicating that *TRI1* is taxonomically informative in *Fusarium* species producing trichothecenes.

**Figure 4:**
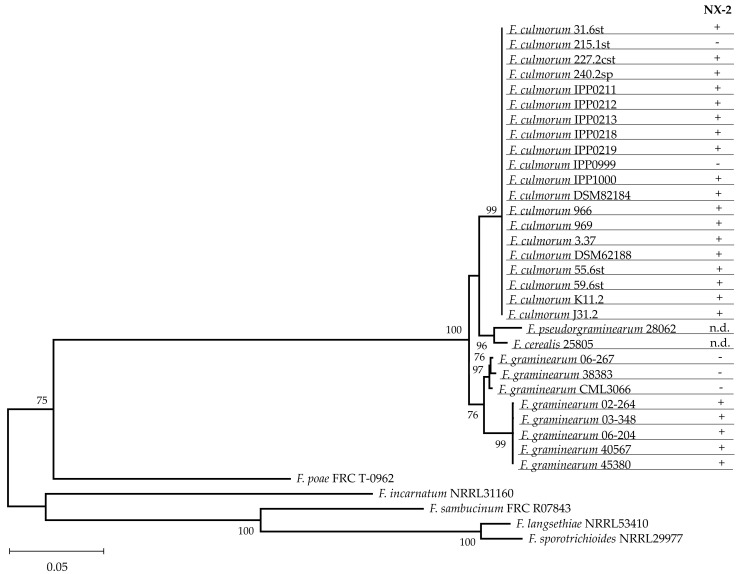
Maximum likelihood estimation of the phylogenetic relationships among the *TRI1* genes in *Fusarium* spp. Complete sequences of the *TRI1* genes of the investigated isolates of *F. culmorum* (see Table 3) and reference sequences were subjected to maximum likelihood analysis [36] assuming the Tamura−Nei model [37]. Bootstrap values (1000 replications) are shown next to the nodes. The production of NX-2 was determined by HPLC-MS/MS; n.d. stands for no data available. Nucleotide sequences were deposited at NCBI with the accession nos. listed in Supplementary Table S3.

The reason for selecting the *TRI1* gene for the analysis was that the product of *TRI1* catalyzes biosynthetic steps distinguishing NX toxins from trichothecenes B, and that polymorphisms in *TRI1* differentiating *F. graminearum* strains producing NX toxins from nonproducers were identified [30]. As expected, all *F. graminearum* strains not producing NX toxins were separated from all strains producing NX toxins. In contrast, no polymorphisms separating *F. culmorum* strains producing NX toxins from nonproducers were found in the *TRI1* gene (Figure 4).

The amino acid sequences of the translation products of the *TRI1* gene, designated Tri1, were identical for all isolates of *F. culmorum* in this study. Isolates of *F. graminearum* producing NX toxins differed from nonproducers in 14 amino acid residues within the heme-binding motif [35] (Table 2). In all *F. culmorum* strains used in this study, comprising 18 producers and 2 nonproducers of NX-2, these amino acid residues were identical, and they matched the corresponding residues in the strains of *F. graminearum* that did not produce NX toxins. Thus, the reason for the production of NX-2 by *F. culmorum* is not an NX-specific form of *TRI1* found in NX-2 producers of *F. graminearum.*

## 3. Discussion

### 3.1. Production of NX-2 Is a Characteristic Feature of F. culmorum 

So far, production of NX-2 has exclusively been reported in *F. graminearum,* which shares a high level of genomic similarity with *F. culmorum*, produces the same mycotoxins, and has the same host range [6,38]. The production of NX-2 was discovered in an atypical population of *F. graminearum* from the Midwest of the USA, named the Northland population, which did not produce any known trichothecene [33]. Producers of NX-2 were later found in this population [30], but only a small fraction of *F. graminearum* strains collected in the area produced NX toxins. A later study on *F. graminearum* in wheat in Ontario (Canada), however, reported that 80% of the investigated strains produced NX toxins [16]. A key finding of the current study is that NX-2 production is not limited to *F. graminearum*, and, contrary to the previous assumption [35], it is not endemic to northern USA and southern Canada.

The ability to produce NX-2 appears to be a common feature of *F. culmorum.* Among the 20 strains of *F. culmorum* isolated in Europe and Asia over a period of 70 years, 18 strains produced NX-2 (Table 1). We suppose that the production of NX-2 toxins in cultures of *F. culmorum* remained unnoticed because NX toxins were not monitored in routine surveys. No commercial standards for NX-2 were available at the time of writing. Because infection of grain crops with *F. culmorum* is widespread, we assume that contamination of grains and grain products with NX-2 might be common.

### 3.2. Both Chemotypes of F. culmorum Produce NX-2

Isolates of *F. culmorum* belong to the 3-ADON and NIV chemotypes; the 15-ADON chemotype is absent [26,27,28,39,40]. Most *F. culmorum* isolates studied in this work belonged to the 3-ADON chemotype and produced NX-2 toxin. The only strain of the NIV chemotype produced NX-2 toxin, too (Table 1). Investigation of further isolates has yet to confirm that this generally holds for strains of the NIV chemotype. In *F. graminearum,* all producers of NX toxins identified in the first report from the USA belonged to the 3-ADON chemotype [30]. Thus, production of NX-2 by a strain of the NIV chemotype is another unique feature distinguishing NX-2 production in *F. culmorum* and *F. graminearum*. Another group studying *F. graminearum* strains from Canada confirmed this finding: NX-2 producers were rare, and all of them (including five isolates from Ontario, see below) were assigned to the 3-ADON chemotype [41]. The most recent study on *F. graminearum* from Ontario reported contradictory results, assigning most NX-2 producers to the 15-ADON chemotype [16]. Production of NX-2 was verified by chemical analysis in both studies. In the study of Kelly et al. [41], the 3-ADON/15-ADON chemotype was assessed by PCR-RFLP while Crippin at al. [16] used both PCR-RFLP and chemical analysis. Incongruencies between the chemotype prediction by PCR-RFLP and the results of chemical analysis, reported by several studies [14,16,29,42], cannot explain the contradictory chemotype assignments mentioned above because both studies used the same PCR assay (*TRI*3/*TRI*12), yet Crippin et al. [16] reported that all their NX-2 producers belonged to the 15-ADON chemotype while Kelly at al. [41] assigned all their NX-2 producers to the 3-ADON chemotype. Crippin at al. [16] also analyzed trichothecenes in cultures on three growth media by HPLC-MS/MS, using a special elution gradient for the separation of 3-ADON and 15-ADON (these mycotoxins often co-elute, and they cannot be reliably distinguished by MS fragmentation). They reported the concentrations of 15-ADON, but unfortunately not the concentrations of 3-ADON and/or DON. 

The acetylation of C-3-OH of NX-2 (Figure 3) is reminiscent of 3-ADON. Varga et al. [30] showed that when part of the translation product of *TRI1* in a strain of the 15-ADON chemotype was replaced with a *TRI1* segment specific for the production of NX-2, the recombinant strain accumulated NX-4 (a derivative acetylated on C-15-OH, reminiscent of 15-ADON) rather than NX-2. The replacement of NX-specific polypeptide in an NX-2 producer with a protein segment from a 15-ADON producer converted the NX-2-producing strain into a 3-ADON producer. Both results strongly support the hypothesis that NX-2 producers in *F. graminearum* developed from strains of the 3-ADON chemotype. The controversy between the assignment of chemotypes to NX-2 producers from Ontario in [41] and [16] remains unresolved. 

### 3.3. Fusarium culmorum Produces NX-2 Simultaneously with DON and 3-ADON or NIV

A search for new trichothecenes in *F. graminearum* that facilitated the discovery of NX toxins was motivated by a failure to detect any trichothecene in a group of atypical strains of *F. graminearum* from the USA [30,33]. These strains produced NX toxins but no trichothecenes B. The recent study from Ontario cited above [16] reported that cultures of *F. graminearum* accumulating NX-2 simultaneously accumulated 15-ADON. 

Cultures of NX-2-producing *F. culmorum* strains in our study accumulated 3-ADON (most strains) or NIV (a single strain). In cultures of the 3-ADON chemotype, NX-2 accumulated at amounts corresponding to 2–8% of 3-ADON, and the content of NX-2 and trichothecenes B was tightly correlated (Figure 1). In contrast, the concentrations of NX-2 to 15-ADON in *F. graminearum* cultures in the study of Crippin et al. [16] were not correlated; the ratio NX-2/15-ADON varied from 0.003 to 33. 

The only strain in our work producing NX-2 at an amount comparable to trichothecenes B was a strain of the NIV chemotype (Table 1). In rice cultures of this strain, NX-2 reached 75% of the concentration of NIV and 66% of total trichothecenes B. Future studies must clarify whether the relatively high production of NX-2 is a typical feature of strains of *F. culmorum* with the NIV chemotype. In *F. graminearum,* no strain of the NIV chemotype has so far been reported to produce NX-2.

### 3.4. NX-2 Production by F. culmorum Is not Caused by a Variant of TRI1 

The reason for the production of NX toxins by certain strains of *F. graminearum* is that these strains harbor a special variant of the *TRI1* gene [30]. The gene, which is only present in genomes of trichothecene-producing *Fusarium* species [43], encodes cytochrome P450 monooxygenase (calonectrin C7/C8 hydroxylase) Tri1, which catalyzes oxidation of C-7 and C-8 [44,45]. The difference between *TRI1* of NX-2 producers and nonproducers allowed a PCR-RFLP assay for NX-2 producers [46]. We have not found such a polymorphism in the *TRI1* gene of *F. culmorum*. 

In *F. graminearum*, changes in Tri1 specific for NX toxins occurred in the heme-binding motif close to the C-terminus. Furthermore, Ramdass et al. [47] found a new potential glycosylation site in the enzyme of NX-2 producers. It was located at a large distance from the heme-binding site, but the authors suggested that glycosylation may modulate enzyme activity via protein folding. None of these changes occurred in the *TRI1* gene of NX-2-producing *F. culmorum*. Some residues in the heme-binding segment differed from corresponding residues in *F. graminearum* (purple in Table 2), but these residues occurred in NX-2 producers and nonproducers. 

Factors other than the amino acid sequence of Tri1 must suppress its activity towards C-8 of calonectrin in *F. culmorum* strains producing NX-2. The enzyme is located inside the endoplasmic reticulum, with two hydrophobic segments close to the ends of the protein crossing the membrane [47]. Other proteins and especially other cytochromes P450, which compete with Tri1 for the same NADPH-cytochrome P450 reductase, share this location. Cytochromes P450 are known to interact with each other and form heterooligomers, which modifies their activity [48]. The interaction of Tri1 with other proteins anchored in the membrane of endoplasmic reticulum may suppresses the oxidation of C-8 in NX-2-producing strains of *F. culmorum*. 

### 3.5. NX-2 and the Aggressiveness of F. graminearum and F. culmorum

In *F. graminearum*, a shift from the 15-ADON to the 3-ADON chemotype observed in the last decades supports the hypothesis that the 3-ADON chemotype is more aggressive. For instance, an increase in the 3-ADON chemotype at locations where 15-ADON producers used to be predominant was reported in a study from North America [49]. Many studies of the population structure in *F. graminearum* elucidated the relationship between genotype, chemotype, and aggressiveness, aiming to explain how selection and gene flow shaped the populations (e.g., [17,20,35,41,46,49,50,51,52]). Field studies documented the success of the 3-ADON chemotype of *F. graminearum,* but they could not address its cause, which requires a biochemical approach. According to a hypothesis from the lab of Gerhard Adam [24], acetylation of C-3-OH prevents detoxification of DON, which is a virulence factor, by plant UDP-glycosyltransferases. In NX-2, the hydroxyl on C-3 is also protected by acetylation, and Varga at al. [30] suggested that the production of NX-2 may benefit *F. graminearum* during the infection in the same way. Glucosylation of DON takes place in the cytoplasm while the target of DON is protein synthesis in rough endoplasmic reticulum, where the acetyl group would have to be removed. This is plausible because the endoplasmic reticulum is rich in hydrolases [53]. Varga et al. [30] also speculated that the lack of carbonyl on C-8 circumvents detoxification by glutathionylation. This hypothesis holds for *F. cumorum,* too. 

Can the effect of NX-2 on the aggressiveness of *F. graminearum* or *F. culmorum* be proved? Field trials with natural isolates are unlikely to generate a conclusive proof. The same situation exists for the claim that the 3-ADON chemotype is more aggressive than the 15-ADON chemotype, which was supported by some studies [20,21,22,23] yet rejected by others [18,19,52]. Field isolates differ in many properties modulating aggressiveness. Some of them are likely linked to the chemotype. Isogenic strains differing only in the gene in question are required. Regarding the relative aggressiveness of the chemotypes 3-ADON and 15-ADON, strains with swapped *TRI8* genes or *TRI8* chimeras, constructed by Alexander et al. [54], could be used. Similarly, infection experiments with *F. graminearum* strains harboring *TRI1* with swapped domains controlling the NX-2 production, constructed by Varga at al. [30], would reveal the effect of NX-2 production on aggressiveness.

### 3.6. Do further Fusarium Species Produce NX Toxins?

Comparison of the amino acid residues in the Tri1 sequence distinguishing NX-2-producing isolates from nonproducers in *F. graminearum* (Table 2) showed that certain residues specific for the production of NX-2 are also present in Tri1 of other *Fusarium* species. For instance, Tri1 in a particular *F. sambucinum* strain shared three residues with NX-2-producing strains of *F. graminearum*, and it differed from Tri1 of strains that did not produce NX-2 in another three positions. We suggest that this species should be examined for NX-2 production. *Fusarium cerealis* and *F. pseudograminearum* harbor *TRI1* genes very similar to *TRI1* of *F. culmorum* (Figure 4), and the amino acid sequence in the heme-binding segment of their Tri1 protein is identical with the corresponding sequence in *F. culmorum* (Table 2). Examination of these species for the production of NX toxins, too, appears worthwhile. A universal PCR-based assay for NX-2 production does not seem feasible.

### 3.7. Can F. culmorum Produce DON and NIV Simultaneously?

It is generally assumed that strains of *F. culmorum* and *F. graminearum* produce either NIV or DON but not both. We believe that this view is biased by the low sensitivity of analytical methods in the past, which only detected trichothecenes at high concentrations (e.g., [27]). Reports of the production of both trichothecenes by a single strain were largely overlooked. For instance, Foroud et al. [3] write in their recent review “NIV chemotypes do not produce DON”. Experimental studies such as [20,55,56], the first two of which are cited in [3], clearly showed simultaneous production of DON and NIV by single strains of *F. culmorum* and *F. graminearum*.

DON and NIV have a common precursor. In NIV producers, the precursor is hydroxylated at C-4 by Tri13 [13]. We suggest that before the entire pool is hydroxylated, some precursor enters the path leading to DON, and thus all cultures producing NIV accumulate small amounts of DON and/or its acetylated derivatives. The presence of NIV in cultures of DON producers can be explained by a residual activity of *TRI*13 or the activities of hydroxylases with a relaxed substrate specificity. In line with this reasoning, cultures of the NIV-producing strain 59.6st in our work accumulated relatively large amounts of DON while cultures of most DON and ADON producers contained small amounts of NIV (Table 1). Similar results were reported in *F. graminearum* (Table 6 in [56]). 

We anticipate that with the widespread use of sensitive analytical methods, small amounts of NIV will often be found in cultures of *Fusarium* strains producing DON, and substantial amounts of DON will be found in cultures of all strains producing NIV. 

## 4. Conclusions

Most strains of *Fusarium culmorum* produce NX-2 toxin simultaneously with deoxynivalenol, 3-acetyldeoxynivalenol, or nivalenol. Strains producing NX-2 do not possess a specific variant of the *TRI1* gene known from NX-2-producing strains of *F. graminearum*. 

## 5. Materials and Methods

### 5.1. Fungal Strains

The strains of *F. culmorum* are listed in Table 3. Strains isolated in the course of this study were obtained from maize grains, rachis, stalks, and oat grains according to Leslie and Summerell [57]. Briefly, samples were surface sterilized for 10 min with 0.1% silver nitrate or 3% sodium hypochlorite, rinsed, and placed on potato dextrose agar (PDA). Isolates were purified via single-spore cultures and grown on PDA for colony characteristics and on low-nutrient agar (SNA, [58]) under long-wave UV light for morphological characterization of spores. For long-term storage, fungal cultures were freeze-dried.

**Table 3:** Strains of *F. culmorum* used in this study.

Species	Origin	Year	Isolate	Host
*F. culmorum*	Germany	2017	31.6st	Maize stalk
*F. culmorum*	Germany	2018	215.1st	Maize stalk
*F. culmorum*	Germany	2018	227.2cst	Maize stalk
*F. culmorum*	Germany	2018	240.2sp	Maize rachis
*F. culmorum*	-	<1984 ^a^	IPP0211 ^b^	Wheat
*F. culmorum*	Italy	<1993 ^a^	IPP0212 ^b^	Wheat
*F. culmorum*	-	1993	IPP0213 ^b^	Barley
*F. culmorum*	Hungary	1991	IPP0618 ^b^	Wheat
*F. culmorum*	Germany	1991	IPP0619 ^b^	Wheat
*F. culmorum*	Germany	2010	IPP0999 ^b^	Wheat
*F. culmorum*	Germany	2010	IPP1000 ^b^	Wheat
*F. culmorum*	Germany	1952	DSM62184 ^c^	Maize grain
*F. culmorum*	Syria	2009–2010	966 ^d^	Wheat
*F. culmorum*	Syria	2009–2010	969 ^d^	Wheat
*F. culmorum*	Germany	2004	3.37 ^b^	Wheat
*F. culmorum*	Germany	1990	DSM62188 ^c^	Maize stalk
*F. culmorum*	Germany	2017	55.6st	Maize stalk
*F. culmorum*	Germany	2017	59.6st	Maize stalk
*F. culmorum*	Germany	2021	K11.2	Oat
*F. culmorum*	Germany	2021	J31.2	Oat

^a^ Isolates were collected before the specified year. ^b^ Isolates of the Plant Phytopathology and Crop Protection Section at the University of Göttingen, Göttingen, Germany. ^c^ Strains were obtained from the German Collection of Microorganisms and Cell Cultures, Braunschweig, Germany. ^d^ The isolates were described by Alkadri et al. [40].

### 5.2. DNA Methods

Fungal DNA was extracted using a CTAB-based protocol [59] from 10 mg of lyophilized mycelium and dissolved in 50 µL of TE (10 mM Tris, 1 mM EDTA, pH 8.0). Segments of marker genes *TEF-1α* and *RPB2* were amplified, and the PCR products subjected to high-resolution melting curve (HRM) analysis as described previously [34]. The *TRI1* gene was amplified for sequencing as four overlapping fragments (Table 4), which were sequenced by the Sanger method and assembled to full-length gene sequences. PCR was carried out in a peqSTAR 96 thermocycler (PEQLAB, Erlangen, Germany) in a total reaction volume of 25 µL. Reaction mixtures were composed of a buffer (10 mM Tris-HCl, 50 mM KCl, 1.5 mM MgCl_2_, pH 8.3 at 25 °C; New England Biolabs, Beverly, MA, USA) adjusted to a 2 mM final MgCl_2_ concentration containing 100 µM of each deoxyribonucleoside triphosphate (Bioline, Luckenwalde, Germany), 0.3 µM of each primer, 0.62 U HotStart-polymerase (New England Biolabs, Beverly, MA, USA), and 1 µL of template DNA solution diluted 100 times. The thermocycling conditions are specified in Supplementary Table S2. PCR products were precipitated with isopropanol, washed with 80% ethanol, and sequenced at the facilities of Macrogen Europe (Macrogen Europe, Amsterdam, The Netherlands).

The sequences were quality-trimmed with Chromas version 2.6.6 (Technelysium Pty. Ltd., South Brisbane, Australia) and assembled to full-length gene sequences; the accession numbers are listed in Supplementary Table S3. Multiple sequence alignments were performed with ClustalW [60] in MEGA version 10.1.8 [36]. Phylogenetic relationships among *TRI1* genes in *Fusarium* spp. were investigated using maximum-likelihood analysis using MEGA X under the assumption of Tamura’s and Nei’s substitution model [37].

### 5.3. Mycotoxin Extraction and HPLC-MS/MS

Rice cultures were prepared in 50-mL Falcon tubes (Sarstedt, Nümbrecht, Germany) by autoclaving 3 g of dry polished rice with 5 mL of tap water. The tubes were inoculated with plugs of PDA (0.5 cm diameter) overgrown with 5-day-old mycelium. Rice medium incubated with agar plugs without mycelium served as a control. The cultures were incubated for 21 days at 25 °C in the dark. Fungal metabolites were extracted by shaking with 30 mL acetonitrile/water/acetic acid (84:15:1 (*v*/*v*/*v*)) overnight. Extracts were dried in a vacuum concentrator (Martin Christ, Osterode am Harz, Germany) and residues re-dissolved in methanol/water (20:80 (*v*/*v*)) as described previously [61]. Mycotoxin analysis was carried out using a 1290 Infinity II HPLC system (Agilent Technologies, Waldbronn, Germany) coupled with a 6460 triple quadrupole detector (Agilent Technologies, Waldbronn, Germany). The separation was performed on a Zorbax Eclipse Plus C18 column, 50 × 2.1 mm with 1.8 µm particle size (Agilent Technologies, Waldbronn, Germany). The column oven temperature was 40 °C. Mobile phase A was water with 0.1% formic acid (*v*/*v*), and phase B was methanol with 0.1% formic acid (*v*/*v*). The gradient was as follows: 0 to 0.2 min, 5% B; 0.2 to 8 min, 5% to 35% B; 8 to 8.5 min, 35% to 98% B; 8.5 to 12 min, 98% B; 12 to 12.5 min, 98% to 5% B; 12.5 to 16 min, 5% B. The calibration curve included 11 concentrations from 0.48 to 500 μg/L. A blank was analyzed after every 7th sample and a quality control standard after every 15th sample. The metabolites were detected in a multiple reaction monitoring (MRM) mode. The acquisition parameters and the limits of detection (LOD) and quantification (LOQ) are listed in Table 5. 

### 5.4. Purification of NX-2 

*F. culmorum* isolate 240.2sp, which produced the largest amounts of NX-2, was cultivated on rice media. The cultures were prepared in 4 l flat penicillin flasks with 120 g of organic polished rice and 270 mL of tap water, which were autoclaved and inoculated with 12 agar-plugs (0.6 cm diameter) from 5-day-old fungal cultures. Rice cultures were incubated at 25 °C for 3 weeks in the dark. NX-2 was extracted with a 5-time excess of methanol/water/acetic acid (90:9:1) by shaking overnight. The extract was concentrated to approximately 15% of its original volume using a rotary evaporator R-100 (Buchi, Flawil, Switzerland). The concentrated extract was partitioned with the same volume of ethyl acetate (EtOAc) three times. Combined EtOAc fractions were dried in a rotary evaporator. The residue was dissolved in methanol/water (30:70) and cleared by centrifugation. The supernatant was subjected to flash chromatography (Sepacore Flash system X10/X50, Buchi, Flawil, Switzerland) equipped with a binary pump (modules C-601/C-605) and a UV detector (module C-635). Metabolites were separated on a reverse-phase column (Chromoband Flash cartridge RP C_18_ ec 40–63 µm, 262 × 37 mm, Machery-Nagel, Düren, Germany). Mobile phase A was water with 0.2% acetic acid; phase B was methanol with 0.2% acetic acid. The gradient elution program was as follows: 0–2 min, 5% B; 2–42 min, from 5% to 50% B; 42–45 min, from 50% to 98% B; 45–55 min, 98% B; 55–58 min, from 98% to 5% B; 58–66 min, 5% B. The flow rate was 50 mL min^−1^. The separation was monitored at 254 nm. Fractions containing NX-2 were collected at 33 min and the solvent was evaporated in a rotary evaporator. The residue was re-dissolved in methanol/water (30:70) and subjected to a preparative-HPLC (PU-2086 plus, Jasco, Gross-Umstadt, Germany) equipped with a preparative reverse-phase column (Nucleodur C18 pyramid, 5 µm, 250 × 21mm, Macherey-Nagel, Düren, Germany) and coupled to a UV/VIS detector (UV-970, Jasco, Gross-Umstadt, Germany). NX-2 was eluted by a gradient of water with 0.2% acetic acid (*v*/*v*) (solvent A) and methanol with 0.2% acetic acid (solvent B) as follows: 0–5 min, 5% B; 5–50 min, from 5% to 50% B; 50–55 min, from 50% to 98% B; 55–70 min, 98% B; 70–75 min, from 98% to 5% B; 75–90 min, 5% B. The injection volume was 3 mL, and the flow rate was 14 mL min^−1^. The separation was monitored at 254 nm. NX-2 was collected at 43 min and solvent was evaporated in a vacuum concentrator (Christ, Osterode am Harz, Germany). NX-2 was further polished on Sephadex LH-20 (Sigma-Aldrich, Darmstadt, Germany) in a 460 × 15 mm column eluted isocratically with methanol at a flow rate of 1 mL min^−1^. The eluent was monitored at 254 nm. 

### 5.5. Nuclear Magnetic Resonance (NMR) Spectroscopy

1D (^1^H and ^13^C) NMR spectra were obtained from Bruker Avance 500 Hz and 2D (^1^H,^13^C-HSQC, ^1^H,^13^C-HMBC, ^1^H,^1^H-COSY) NMR spectra were obtained from an Agilent DD2 400 MHz system. The spectra were recorded at 500/400 MHz (^1^H) and 126/101 MHz (^13^C). Chemical shifts were referenced to internal TMS (δ_H_ 0, ^1^H) or MeOH-d_4_ (δ_c_ 49.0, ^13^C). The data are provided as Supplementary Figures S1–S5. 

## Figures and Tables

**Table 1:** Accumulation of trichothecenes in rice cultures of *F. culmorum* strains.

Isolate	Mycotoxin Content (mg/kg)				
	NIV	DON	15-ADON	3-ADON	NX-2
31.6st	0.04	25.6	<LOD	156	3.2
215.1st	<LOD ^a^	4.2	<LOD	90.4	2.4
227.2cst	<LOD	0.5	<LOD	2.1	<LOD
240.2sp	0.38	355	<LOD	552	35.6
IPP0211	0.31	566	<LOD	412	12.2
IPP0212	0.10	162	<LOD	463	16.4
IPP0213	0.33	639	<LOD	388	9.4
IPP0618	<LOD	20.3	<LOD	175	3.7
IPP0619	<LOD	15.9	<LOD	272	14.4
IPP0999	<LOQ ^b^	<LOD	<LOD	<LOD	<LOD
IPP1000	<LOD	12.6	<LOD	132	2.7
DSM62184	0.05	108	<LOD	173	13.5
966	<LOD	25.7	<LOD	118	3.2
969	<LOQ	14.3	<LOD	147	3.2
3.37	<LOD	9.0	<LOD	68.6	1.4
DSM62188	<LOQ	46.5	<LOD	294	13.6
55.6st	<LOQ	31.3	<LOD	85.9	1.2
59.6st ^c^	13.6	0.4	0.9	0.63	10.3
K11.2	<LOD	1.0	<LOD	46.3	1.2
J31.2	0.08	127	<LOD	447	18.6

^a^ LOD = limit of determination; ^b^ LOQ = limit of quantification; ^c^ Strain 59.6st also accumulated fusarenon X at 69 mg/kg.

**Table 2:** Amino acid residues specific for the production of NX-2 in the translation product of *TRI1* of *F. graminearum* and the corresponding residues in *F. culmorum.* Species-specific positions are highlighted in purple for *F. culmorum* and blue for *F. graminearum*. Positions reported to distinguish NX-2-producing strains of *F. graminearum* [35] are marked red.

	Position of Amino Acid Residue																	
Location in *TRI1*	3	5	8	33	35	100	115	134	210	252	254	256	346	361	373	418	430	450
*F. culmorum* NX+ (*n* = 18) ^a^	**I**	**S**	**S**	A	**K**	S	F	**G**	S	R	L	T	F	I	D	Q	T	A
*F. culmorum* NX− (*n* = 2) ^b^	**I**	**S**	**S**	A	**K**	S	F	**G**	S	R	L	T	F	I	D	Q	T	A
*F. graminearum* NX+ (*n* = 3) ^c^	**L**	**T**	**Q**	**T**	**Q**	**N**	**L**	**A**	**T**	**S**	**M**	**N**	**I**	**F**	**E**	**K**	**P**	**V**
*F. graminearum* NX− (*n* = 3) ^d^	**L**	**T**	**Q**	A	**Q**	S	F	**A**	S	R	L	T	F	I	D	Q	T	A
*F. cerealis* 25805	I	S	S	A	K	S	F	G	S	R	L	T	F	I	D	Q	T	A
*F. pseudograminearum* 28062	I	S	S	A	K	S	F	G	S	R	L	T	F	I	D	Q	T	A
*F. poae* FRC T-0962	I	D	P	**T**	K	R	F	A	S	N	L	**N**	F	V	D	Q	T	A
*F. incarnatum* NRRL31160	M	N	N	A	K	K	F	A	S	H	L	D	K	V	D	D	T	A
*F. sambucinum* FRC R-07843	L	D	S	R	K	**N**	F	A	**T**	R	L	**N**	K	V	D	Q	T	A
*F. langsethiae* NRRL53410	F	D	E	R	K	**N**	F	A	A	R	L	**N**	K	V	D	L	T	A
*F. sporotrichioides* NRRL29977	F	D	E	R	K	**N**	F	A	A	R	L	**N**	K	V	D	Q	T	A

^a^ All isolates of *F. culmorum* that produced NX-2 (Table 1); ^b^ Isolates 227.2cst and IPP0999. ^c^ Isolates 06-204, 02-264, and 03-348. ^d^ Isolates PH-1, 06-627, and 38383. *F. graminearum* and *F. culmorum* sequences are highlighted grey. The accession numbers are listed in Supplementary Table S3.

**Table 4:** Oligonucleotides used for the sequence analysis of the gene *TRI1*.

Primer Name	Primer Sequence (5′–3′)	Location ^a^	Source
*TRI1*6IF1	GCCTSATAGCGACGATCTTGC	0–485	[30]
*Tri1*_5prime_RV	GACCGTGAATAACCTCCTTGATCAGT		This study
*Tri1*_5prime_FW	ACTGATCAAGGAGGTTATTCACGGTC	460–1225	This study
*Tri1*_SH_R	CGCTGTCGAGAAGGAACATCTTG		This study
*Tri1*_SH_F	GGCTATGTACAAGATGTTCCTTCTCG	1194–1753	This study
Fg*TRI1*-R1	AACAAGTGGCGAGATCAAACC		[30]
Fc*Tri1*F	ATGGCTATCATCAGCAG	1–1745	This study
*Tri1*R	CAATTCCAATCGCAGACAA		[46]

^a^ Location of the PCR product within the nucleotide sequence of the gene *TRI1* (1753 bp).

**Table 5:** Parameters for HPLC-MS/MS analysis of trichothecenes.

Toxin	Ioniz. Mode	Parent Ion [*m/z*]	Fragm. Voltage [V]	Collision Energy [V]	Product Ion [*m/z*] ^c^	LOD ^b^ [mg/kg]	LOQ ^b^ [mg/kg]
NIV	Pos	313.1	93	7	205.0	0.01	0.03
				15	175.0		
DON	Pos	297.1	100	4	249.1	0.04	0.13
				64	91.2		
3-ADON	Pos	339.2	100	8	231.1	0.02	0.08
				8	203.0		
15-ADON	Pos	339.2	90	10	321.1	0.07	0.23
				10	261.0		
				10	137.0		
NX-2 ^a^	Pos	325.2	90	10	247.2	0.04	0.12
				10	229.2		
				10	199.1		
				25	121.2		
				35	105.1		

^a^ Analytical reference for NX-2 was kindly provided by Dr. Franz Berthiller (BOKU, Vienna, Austria). ^b^ Limit of detection (LOD) and limit of quantification (LOQ) were calculated based on the standard deviation of the blank [62]. ^c^ *m*/*z* of product ions used for quantification are underlined.

## Data Availability

Data is contained within the article or supplementary materials. DNA sequences were desposited in NCBI; their accession nos. are listted in Supplementary Table S3.

## Supplementary Materials

The following supporting information can be downloaded at: https://www.mdpi.com/article/10.3390/toxins14070456/s1, Figure S1: ^1^H NMR spectrum of NX-2; Figure S2: ^13^C NMR spectrum of NX-2; Figure S3: ^1^H, ^13^C HSQC NMR spectrum of NX-2; Figure S4: ^1^H, ^13^C HMBC spectrum of NX-2; Figure S5: ^1^H, ^1^H COSY spectrum of NX-2. TableS1: Comparison of NMR spectra of NX-2 with metabolite purified from *F. culmorum* 240.2. Table S2: Conditions used for the amplification of *TRI1* gene. Table S3: Acc. nos. of sequences of the *TRI1* gene.
