# Supplementary material for: Fusarium culmorum Produces NX-2 Toxin Simultaneously with Deoxynivalenol and 3-Acetyl-Deoxynivalenol or Nivalenol
Source: Toxins (Basel). 2022 Jul 2;14(7):456. doi: 10.3390/toxins14070456 (PMC9324393; doi:10.3390/toxins14070456)
Supplement: Supplementary file 1 [file toxins-14-00456-s001.zip › toxins-1733919-supplementary.pdf]

toxins

MDPI

Article

Fusarium culmorum Produces NX-2 Toxin Simultaneously with Deoxynivalenol and 3-Acetyl-Deoxynivalenol or Nivalenol

Simon Schiwek, Mohammad Alhussein, Charlotte Rodemann, Tuvshinjargal Budragchaa, Lukas Beule, Andreas von Tiedemann [3] and Petr Karlovsky

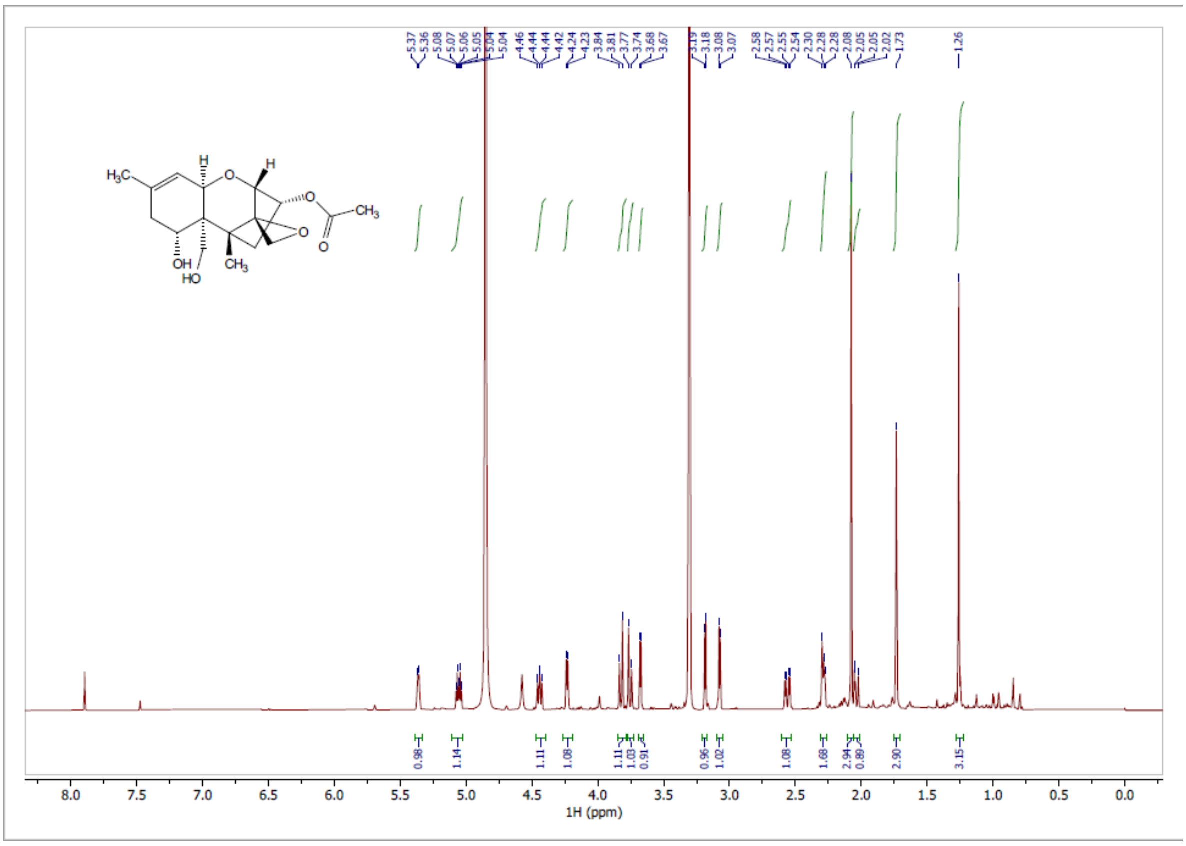

Figure S1. ^{1}H NMR spectrum of NX-2.

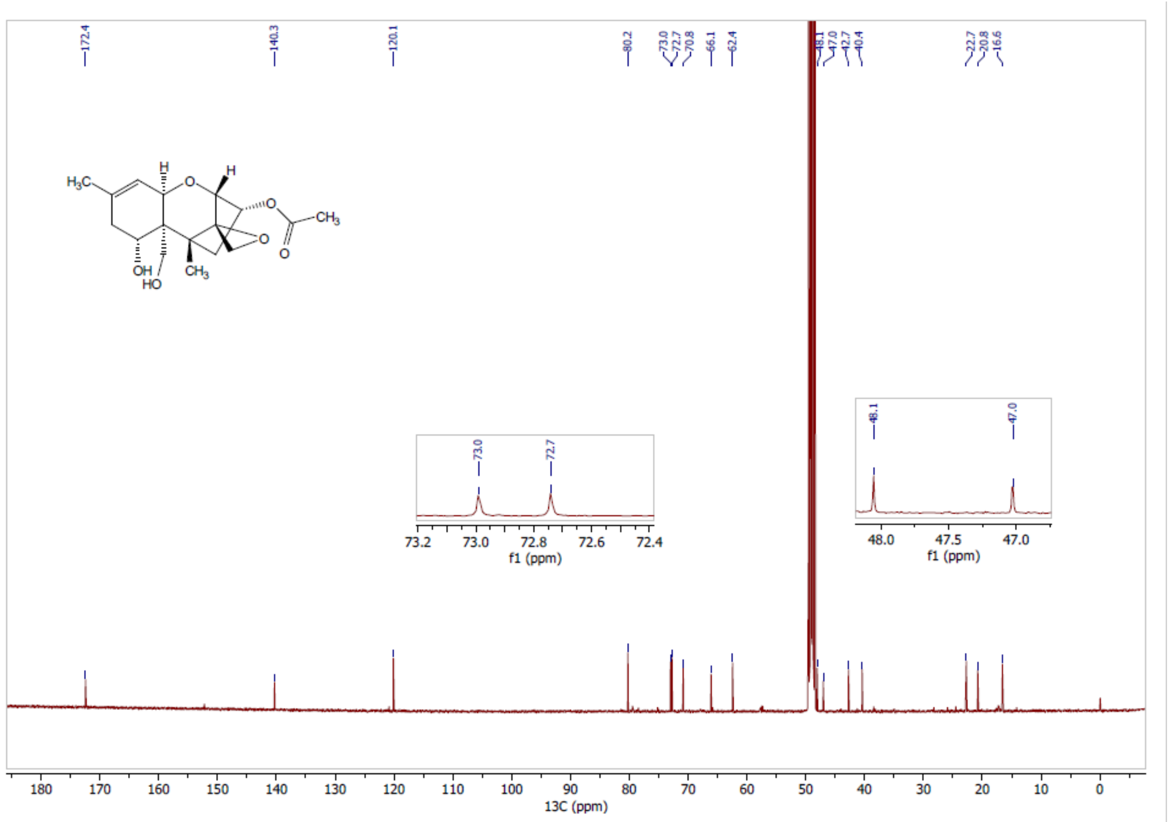

Figure S2. ^{13}C NMR spectrum of NX-2.

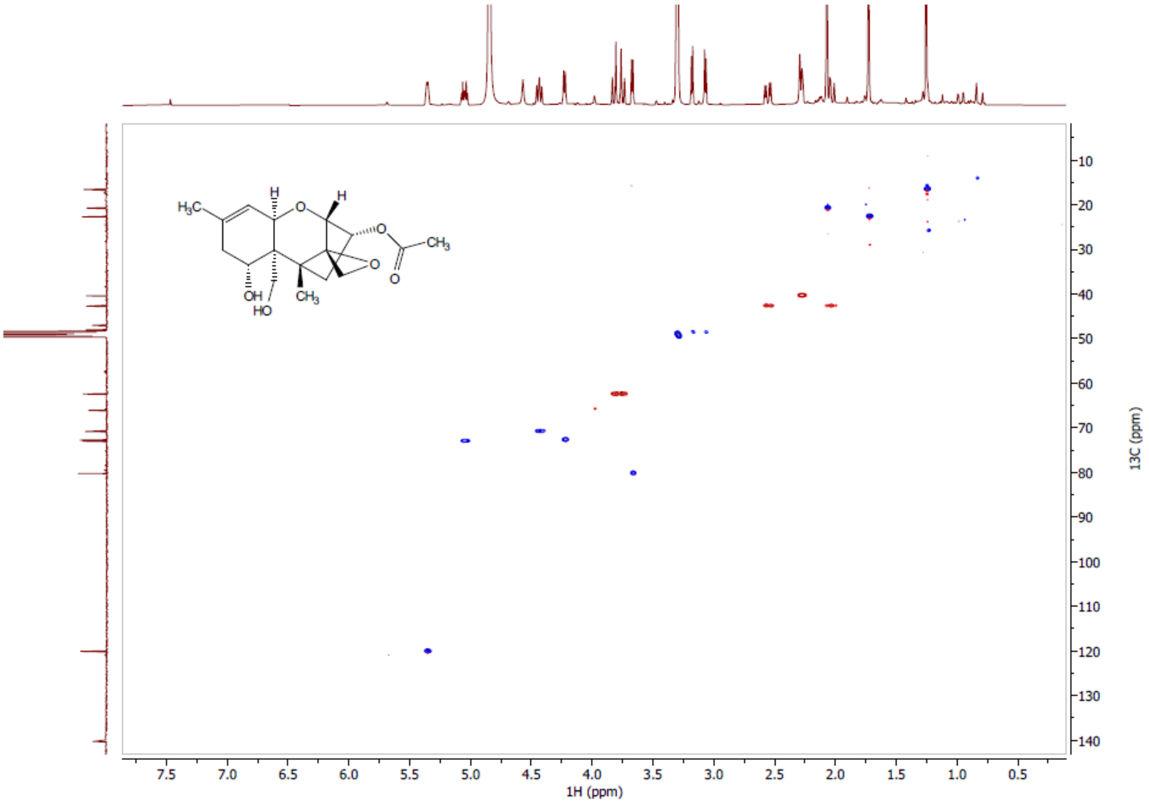

Figure S3. ^{1}H, ^{13}C HSQC NMR spectrum of NX-2.

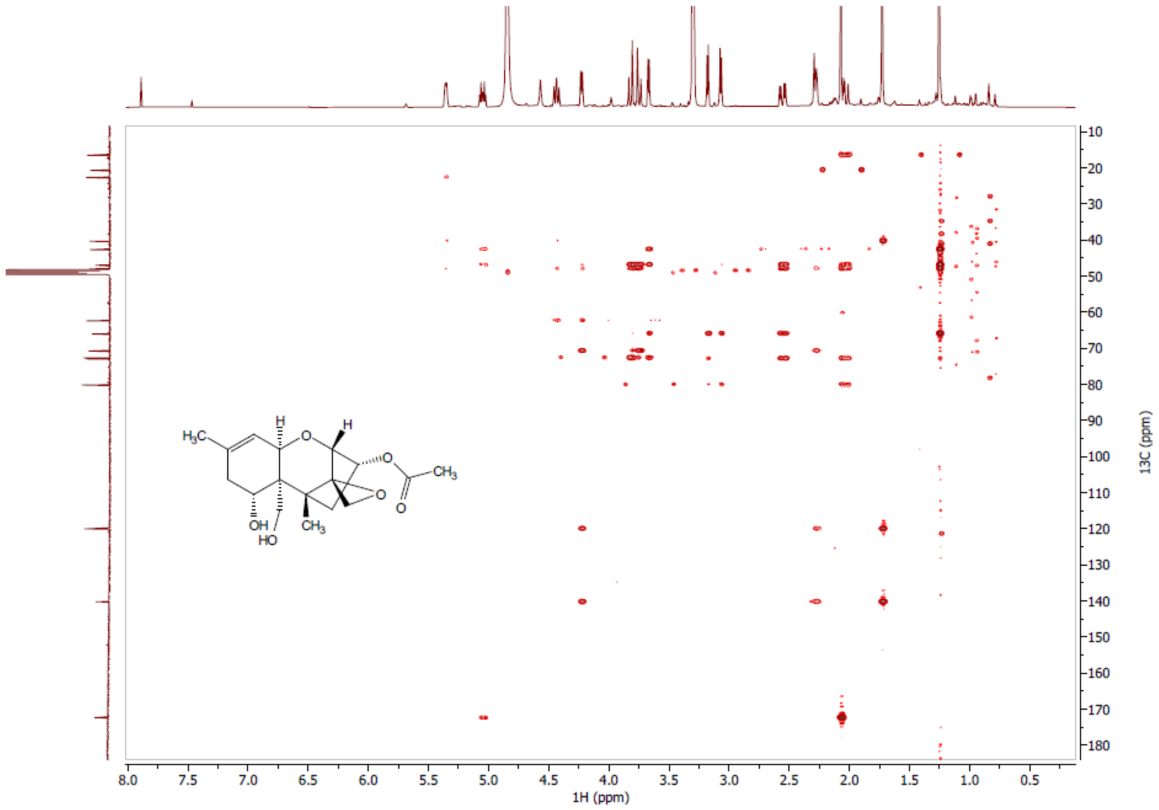

Figure S4. ^{1}H, ^{13}C HMBC spectrum of NX-2.

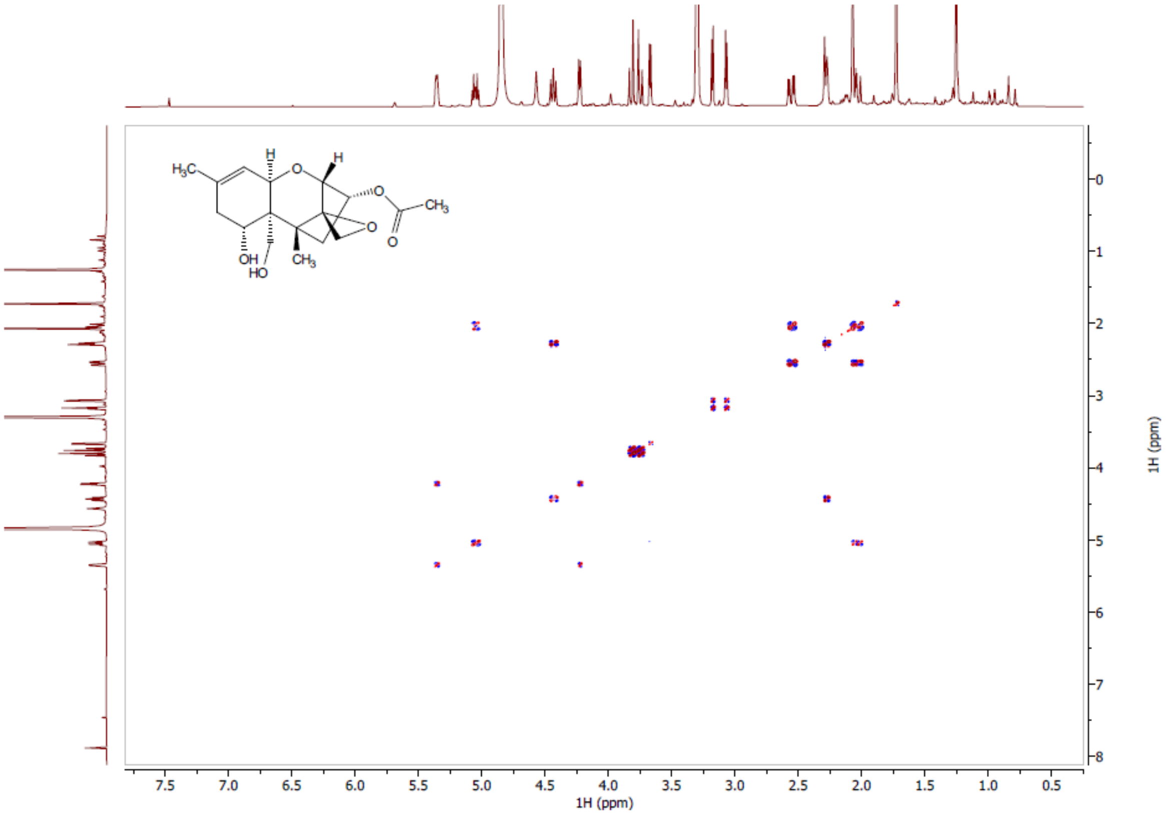

Figure S5. ^{1}H, ^{1}H COSY spectrum of NX-2.

Table S1. Comparison of published NMR spectral data for NX-2 with experimental data obtained on putative NX-2 from rice culture of *F. culmorum* 240.2.

Position	Reported by Varga *et al.*, 2015		Experimental data	
	δ_C	δ_H, mult. (J in Hz)	δ_C	δ_H, mult. (J in Hz)
2	80.3	3.68 d (4.4)	80.2	3.68 d (4.4)
3	73.1	5.06 dt (11.4, 4.4)	73.0	5.06 dt (11.3, 4.4)
4	42.9	2.56 dd (14.9, 4.4)<br>2.05[a] m	42.7[b]	2.56 dd (14.9, 4.4)<br>2.06 – 1.99 m
5	47.2	-	47.0[b]	-
6	48.2	-	48.1	
7	71.0	4.44 dd (9.0, 7.5)	70.8[b]	4.44 dd (9.0, 7.4)
8	40.6	2.32–2.25 m	40.4[b]	2.32–2.25 m
9	140.4		140.3	-
10	120.2	5.36[a] m	120.1	5.36 d (5.4)
11	72.9	4.23 d (5.4)	72.7[b]	4.23 d (5.4)
12	66.2	-	66.1	-
13	48.8	3.19 d (4.3)<br>3.08 d (4.3)	48.6[b]	3.18 d (4.4)<br>3.08 d (4.4)
14	16.7	1.26 s	16.6	1.26 s
15	62.6	3.83 d (11.5)<br>3.76 d (11.5)	62.4[b]	3.82 d (11.6)<br>3.76 d (11.6)
16	22.9	1.74 s	22.7[b]	1.73 s
17 (3-Ac)	172.6	-	172.4[b]	-
18 (3-Ac)	20.9	2.08 s	20.8	2.08 s

[a] Misprint in [Varga et al.: Environ. Microbiol. 2015, 17:2588]; [b] All 13C peaks are shifted 0.1 ppm upfield; a 0.2 ppm difference comes from rounding the second decimal.

Table S2. Conditions used for the amplification of the *TRI1* gene of *F. culmorum.*

Forward primer	Reversed primer	Thermal cycling (identical for all 4 reactions)	No. of cycles	Length [bp]
TRI16IF1	Tri1_5prime_RV	Denatuation: 94 °C, 30 s	35	1641
Tri1_5prime_FW	Tri1_SH_R	Annealing: 59 °C, 30 s	35	764
Tri1_SH_F	FgTRI1-R1	Elongation: 68 °C, 120 s	35	760
FcTri1F	Tri1R	Term. elong.: 68 °C, 5 min	35	1745

Table S3. Accession numbers of sequences of the *TRI1* gene.

Species	Isolate	Accession No.
F. culmorum	K11.2	OM144918
F. culmorum	J31.2	OM144919
F. culmorum	IPP1000	OM144920
F. culmorum	IPP0999	OM144921
F. culmorum	IPP0619	OM144922
F. culmorum	IPP0618	OM144923
F. culmorum	IPP0213	OM144924
F. culmorum	IPP0212	OM144925
F. culmorum	IPP0211	OM144926
F. culmorum	DSM62188	OM144927
F. culmorum	969	OM144928
F. culmorum	966	OM144929
F. culmorum	59.6st	OM144930
F. culmorum	55.6st	OM144931
F. culmorum	31.6st	OM144932
F. culmorum	DSM62184	OM144933
F. culmorum	3.37	OM144934
F. culmorum	240.2sp	OM144935
F. culmorum	227. 2cst	OM144936
F. culmorum	215.1st	OM144937
F. graminearum	CML3066	LT222053
F. graminearum	06-267	KX183401
F. graminearum	38383	KX183278
F. graminearum	06-204	KM999943
F. graminearum	02-264	KM999941
F. graminearum	03-348	KM999942
F. graminearum	40567	KX183282
F. graminearum	45380	KX183296
F. cerealis (*F. crookwellense*)	25805	KX183232
F. pseudograminearum	28062	KX183238
F. langsethiae	NRRL53410	HQ594538
F. sporotrichioides	NRRL29977	HQ594536
F. incarnatum	NRRL31160	GQ915526
F. sambucinum	FRC R-07843	GQ915521
F. poae	FRC T-0962	GQ915520
